# In Spite of Curative Radical Pulmonary Procedures, Lesser Pulmonary Resection Shows More Favorable Prognosis in Surgically Treated NSCLC With Synchronous Isolated Cranial Oligometastases

**DOI:** 10.3389/fsurg.2021.645870

**Published:** 2021-02-25

**Authors:** Erkan Kaba, Eyüp Halit Yardımcı, Jahnavi Kakuturu, Alper Toker

**Affiliations:** ^1^Department of Thoracic Surgery, Group Florence Nightingale Hospitals, Demiroglu Bilim University, Istanbul, Turkey; ^2^Department of Cardiovascular and Thoracic Surgery, West Virginia University Heart & Vascular Institute, Morgantown, WV, United States

**Keywords:** lung cancer, oligometastases, cranial, synchronous, non-small cell, pulmonary resection, radiotherapy, multimodality treatment

## Abstract

Oligometastatic disease in lung cancer is not a rare condition as previously thought. Among 812 non-small cell lung cancer patients treated surgically with lung resection between October 2011 and October 2018 at the Department of Thoracic Surgery, Florence Nightingale Hospitals, Turkey, 28 patients (3.4%) had synchronous cranial metastases. We analyzed synchronous isolated cranial metastases patients treated by locally ablative treatments (surgery, radiotherapy, or both). Metastases existing at the diagnosis of primary cancer were considered as synchronous, and their treatment was performed before (at least 1 month) or after (for maximum 1 month) surgery of the primary lung lesion. Prognostic factors affecting survival are evaluated retrospectively to identify clinical factors predicting survival in an effort to better select patients for surgery. Patients having T1-T2 primary lung tumors, no mediastinal lymph node metastasis, receiving minor anatomical lung resection, receiving neoadjuvant chemotherapy, having single cranial metastasis, and receiving surgical cranial metastasectomy were found to have better survival. According to tumor histology, having adenocarcinoma, and not having lymphovascular or visceral pleura invasion correlated with better survival. Average survival time was 52.1 months and median survival was 32 months. The last mortality during the follow-up was at 24 months; cumulative survival was 48.3% at that time. Our study was designed to define the criteria for patients with oligometastatic disease who may benefit from lung resection.

## Introduction

Almost half of patients diagnosed with non-small cell lung carcinoma (NSCLC) have distant metastases at presentation (1). The primary treatment for most patients with metastatic NSCLC is palliative chemotherapy, which results in median survival (MS) of 8–11 months (2). Moreover, with the recent remarkable advances in chemotherapy drugs and strategies, the reported survival of NSCLC patients with distant metastasis has been improving (3), especially for the patients with adenocarcinoma that harbors sensitive driver oncogene mutations or fusion, such as epidermal growth factor receptor (EGFR) (4) and anaplastic lymphoma kinase (ALK) (5). However, several series since the 1980s have reported prolonged survival after complete resection of the primary tumor and secondary lesions in selected patients who presented with only a limited number of metastases, the so-called oligometastatic disease (6).

Focusing on NSCLC, oligometastatic disease (synchronous or metachronous) has been found in 26% of metastatic patients (7). The reported overall survival in oligometastatic NSCLCs patients exceeded the expected 5-year survival observed in historical cohorts of stage IV NSCLC patients (36.8 vs. 4–6%) (8, 9). This group represent a questioning subset of patients for whom the effectiveness of surgery is difficult to evaluate because of high variability among clinical studies and patient selection bias (6). This study is designed to evaluate the following prognostic factors for better survival periods: primary tumor histology and subtypes, primary tumor stage, type of lung resection, presence of mediastinal lymph node metastases, number of cranial metastases, choice of cranial local therapy, and status of systemic therapy. Even though only a small number of patients could receive immunotherapy among surgically treated patients, future studies will have better survival results if more efficient systemic control will be reached with future developments, which may be a reason to consider larger resections than lobectomy without increasing morbidity in selected patients. The purpose of this study was to review our experience with NSCLC patients with extra-thoracic synchronous solitary metastasis who underwent lung surgery with curative intent and to revisit the variables of prognostic value. Further analyses aimed to identify clinical factors predicting survival in an effort to better select patients for surgery.

## Materials and Methods

We retrospectively reviewed clinical and pathologic records of non-small cell lung cancer (NSCLC) patients who underwent pulmonary resection with curative intent by segmentectomy or greater anatomical lung parenchymal resection between October 2011 and October 2018 at the Department of Thoracic Surgery, Florence Nightingale Hospitals, Turkey. Permissions to access and analyze patients' data were taken from Demiroglu Bilim University Ethics Committee. Among 812 lung cancer patients treated surgically with lung resection, 28 patients (3.4%) had synchronous cranial metastases. We analyzed synchronous isolated cranial metastases patients treated by locally ablative treatments (surgery, radiotherapy, or both). Metastases existing at the diagnosis of primary cancer were considered as synchronous, and their treatment was performed before (at least 1 month) or after (for maximum 1 month) surgery of the primary lung lesion.

A histologic analysis of the tumor was conducted according to the World Health Organization classification for cell types (10). The clinical or pathologic stage of the disease was defined based on the general rules for the TNM Classification of Malignant Tumors (8th edition) (11). The eligibility for surgical resection in this series of patients was determined based on clinical practice. This included patients with an Eastern Cooperative Oncology Group performance status (PS) of 0–1, an estimated postoperative forced expiratory volume in 1 second (FEV1) >800 ml/m^2^ of the body surface area or 40% FEV1, and adequate preserved organ functions expected to tolerate chemotherapy, radiotherapy, or both. Selective patients who had lower lung functions with <3 cm tumors were included and received segmentectomy. Patients were evaluated by physical examination, contrast-enhanced computed tomography (CT) of the chest, contrast-enhanced magnetic resonance imaging (MRI) of the brain, fluorodeoxyglucose positron emission tomography (PET/CT), and flexible optical bronchoscopy (FOB). In patients with marginal physiological status, decision of operability was made on an individual basis, according to results of diffusion capacity of the lung for carbon monoxide (DLCO), and an exercise test (stair climbing or 6-min walking test). All patients underwent preoperative echocardiography with assessment of left ventricular ejection fraction and assessment of systolic pulmonary pressure. Patients with ejection fraction (EF) under 40%, and systolic pulmonary artery pressure (PAP) above 45 mmHg were accepted as physiologically inoperable. The patients who had other concomitant uncontrolled malignancies or serious comorbidities, such as clinically significant cardiac disease, active infection, or psychiatric disorders did not undergo surgical resection.

Segmentectomy, lobectomy, or pneumonectomy with radical mediastinal lymph node dissection was performed through a thoracotomy, video assisted thoracoscopic surgery (VATS) or robot assisted thoracic surgery (RATS) in all patients. The principles of surgical resection were en bloc removal of the affected lobe or lung parenchyma with adjacent structures, and systemic hilar and mediastinal lymph node dissection. Resection was not abandoned due to the need for pneumonectomy in patients with low expected morbidity. Preoperative mediastinoscopy or endobronchial ultrasonography (EBUS) was performed routinely in all patients. Mediastinoscopy was performed in the same session in cases receiving cranial operation first. Lung resection was not performed in patients with pathologically proven N2 involvement at endobronchial ultrasound or mediastinoscopy.

All cases were discussed preoperatively at Radiology Council for determination of resectability and for the presence of additional nodule. Decisions about treatments of lung cancer at our institution are taken on an individual-based discussion during a weekly Multidisciplinary Tumor Board. Institutional policy is to consider locoregional treatments for oligometastatic disease only in cases where the number of metastatic lesions is 3 or less, and consists of adrenal and cranial metastases, because only these sites are considered to be amenable to local ablative therapy as part of the initial management of oligometastatic lung cancer. Treatment of brain metastases consisted of surgery, stereotactic radiosurgery (SRS), whole-brain radiotherapy (WBRT), or a combination of both. The choice was made according to radiological localization of lesions, and patient's performance status. First choice was surgery if possible, followed by SRS to tumor cavity. In recent years, radiation oncologists chose to perform SRS over WBRT after surgery. Dexamethasone (16 mg) and Levetiracetam (2 × 500 mg) therapy was initiated per oral as antiedema therapy regimen and continued for 1 month. Chemotherapy was essentially a part of the global management. It was administered in a neoadjuvant or adjuvant setting; neoadjuvant was the preferred treatment modality, but adjuvant therapy was an option in some cases (e.g., thoracic surgery considered urgent because of uncontrolled symptoms or topography of the lesion). Standard algorithm of our clinic for oligometastatic lung cancer patients started with treatment of the cranial lesion(s), cranial local therapy (surgery, SRS, WBRT), followed by lung resection surgery. Adjuvant chemotherapy was preferred after local treatments. All patients either with clinically N2 disease or not underwent EBUS or mediastinoscopy or both. T4 lung tumor patients received chemotherapy and restaging prior to surgery.

Patient characteristics, treatment procedures, and short-term outcomes were collected using a standardized case report form. Data collection included use of induction chemotherapy, type of resection, histologic type of tumor, main diameter of tumor, and presence of lymphovascular emboli or perineural spreading. The presence of vascular or lymphatic tumor emboli was assessed by standard hematoxylin and eosin staining on samples from tumor and adjacent non-tumoral lung tissue and defined as the presence of aggregates of tumor cells inside vascular or lymphatic microvessels. Main outcome measures analyzed were 30- and 90-day mortality of surgery (brain and lung) radiotherapy, and overall survival. The institutional review board and ethics committee reviewed and approved the protocol of this retrospective analysis. Written informed consent was obtained from all patients.

## Statistical Analysis

SPSS (Statistical Package for Social Sciences) for Windows 24.0 program was used for statistical analysis. Kaplan-Meier analysis and Log Rank testing were used in survival analyses as well as descriptive statistical methods (mean, standard deviation, frequency) when assessing the study data. The results were evaluated in the 95% confidence range and the statistical significance was *p* < 0.05.

## Results

The characteristics of the 28 patients included in the analysis are listed in Table 1. The median age of the patients was 64 years (range, 46–76 years). There were 21 men and 7 women. At the time of the initial treatment, 12 patients (42.9%) were Eastern Cooperative Oncology Group PS 0, and 16 (57.1%) were PS 1. Pathologic diagnosis of the primary tumor was mandatory, and the major histologic type was adenocarcinoma in 19 patients (67.9%), followed by squamous cell carcinoma in 5 patients (17.9%). Eight patients had a tumor status of pT1, 9 had pT2, 4 had pT3, and 7 had pT4. Twenty patients had a lymph node status of pN0, 4 had pN1, and 4 had incidental pN2. Twenty-five patients had single metastatic lesion, 1 patient had two and 2 patients had three metastatic lesions.

**Table 1:** Patient characteristics table.

Age*; mean ± SD (min–max)*		62.32 ± 8.98 (46–76)
Gender*; n (%)*	Female	7 (25.0)
	Male	21 (75.0)
Follow-up period *(months); mean ± SD (min–max)*		25.71 ± 23.47 (4–92)
Mortality; *n (%)*	Alive	16 (57.1)
	Ex	12 (42.9)
Tumor diameter *(cm); mean ± SD (min–max)*		4.51 ± 2.85 (1.1–14)
T Stage; *n (%)*	T1	8 (28.6)
	T2	9 (32.1)
	T3	4 (14.3)
	T4	7 (25.0)
N Stage; *n (%)*	N0	20 (71.4)
	N1	4 (14.3)
	N2	4 (14.3)
Surgical procedure; *n (%)*	Segmentectomy	4 (14.3)
	Lobectomy	13 (46.4)
	Pneumonectomy	4 (14.3)
	Extended resection	7 (25.0)
Anatomic resection Site; *n (%)*	R Segmentectomy	4 (14.3)
	RU Lobectomy	7 (25.0)
	RL Lobectomy	6 (21.4)
	LU Lobectomy	4 (14.3)
	LL Lobectomy	3 (10.7)
	L Pneumonectomy	4 (14.3)
Surgical technique; *n (%)*	Thoracotomy	13 (46.4)
	VATS	8 (28.6)
	RATS	7 (25.0)
Pathology; *n (%)*	Adenocarcinoma	19 (67.9)
	Squamous cell ca	5 (17.9)
	Adenosquamous ca	1 (3.6)
	Large cell ca	2 (7.1)
	Sarcomatoid ca	1 (3.6)
Cranial metastases number; *n (%)*	1	25 (89.3)
	2	1 (3.6)
	3	2 (7.1)

All 28 patients underwent curative intent pulmonary resection by lobectomy (13 of 28; 46.4%), segmentectomy (4 of 28; 14.3%), pneumonectomy (4 of 28; 14.3%), or extended resection (7 of 28; 25%), with systemic mediastinal lymph node dissection. All pneumonectomies were left pneumonectomy. One of the patients had completion pneumonectomy at postoperative day 1 because of bronchial stenosis after sleeve lobectomy. Other 3 patients had central tumors. Extended resection included right upper sleeve lobectomy (*n* = 1), partial vertebra resection (*n* = 1), bi-lobectomy (*n* = 1), right upper double sleeve resection (*n* = 1), and chest wall resection (*n* = 3). Lung resection was performed with VATS in 8 patients, RATS in 7 patients, and thoracotomy in 13 patients. Morbidity occurred in 14 patients (50%). Prolonged air leak was the commonest complication (50%), followed by arrhythmia (10.7%), and pneumonia (7.1%). The mean length of hospital stay was 6.4 (range, 4–15) days. Morbidity was not related with age, type of operation, presence of comorbidities, and requirement of extended resections. Not any significant relationship between the respiratory function capacities of the patients and the survival rates was observed in the multivariate analysis.

Local treatments of cranial metastasis consisted of surgery in 16 (57.1%) patients, and surgical removal was integrated with postoperative SRS to the surgical cavity afterwards. In patients treated with radiotherapy alone (*n* = 12, 42.9%), treatment consisted of SRS therapy or WBRT. There were no recorded deaths at 30- and 90-day following lung or cranial surgery.

**Figure 1:**
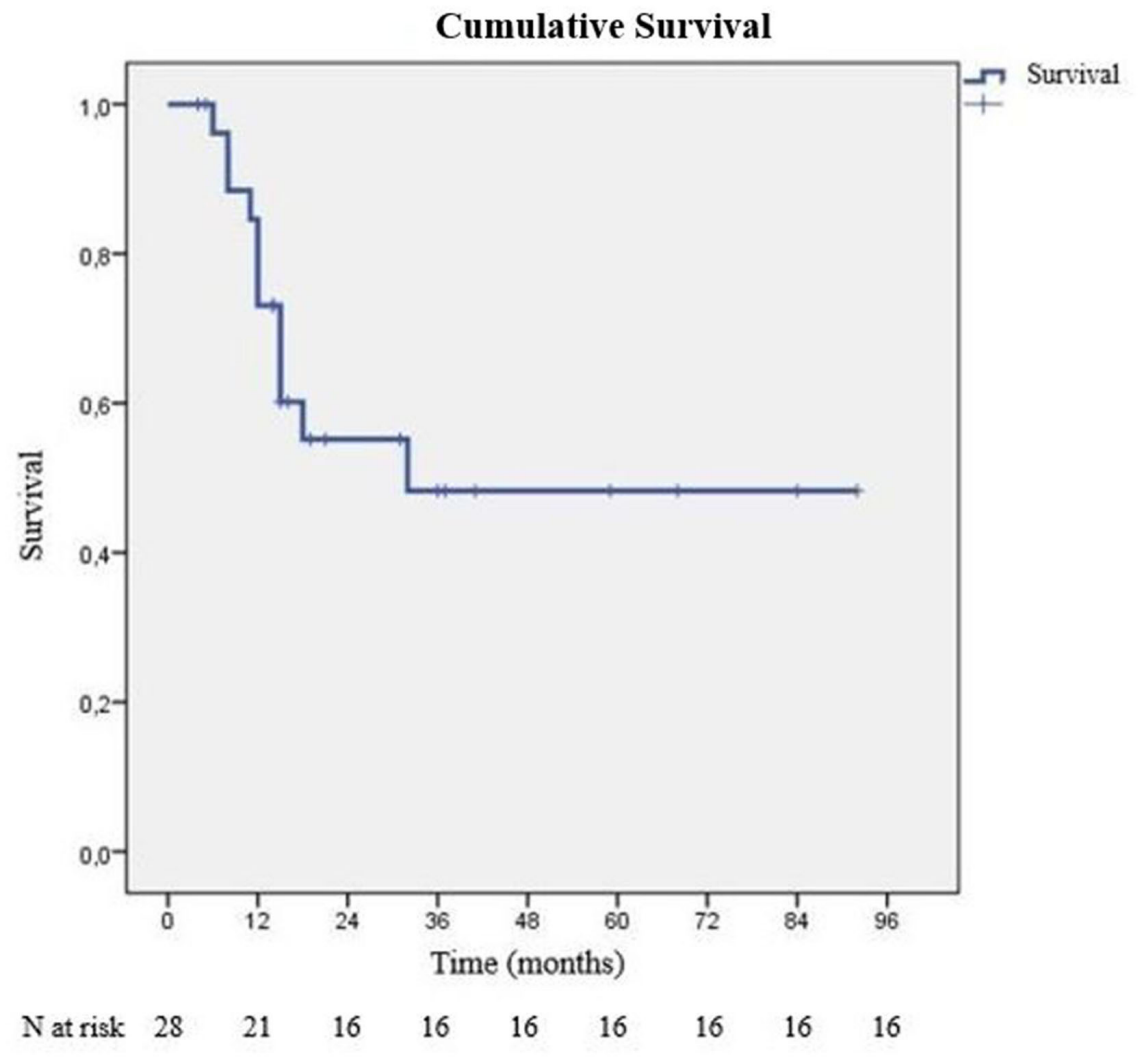
Overall survival graph.

During our follow-up period, 16 of 28 patients survived. Average survival time was 52.10 ± 8.32 months, median survival time was 32 months. The last mortality during the follow-up was at 24 months. At the end of first year, cumulative survival (CS) was 60.2% (SD: 9.8%), and at the second year, it was 48.3% (SD: 11%). After the second year, no additional mortality was observed, and CS percentage stayed the same as 48.3% (Figure 1). There were 6 patients who died of cancer within 1 year of starting treatment. One patient died with local recurrence (intrathoracic), another patient with cranial recurrence, and the remaining patients died due to disseminated metastatic disease and general condition disorders after the initiation of chemotherapy. Survival differences according to inspected criteria are listed in Table 2.

**Table 2:** Survival between subgroups table.

		***N***	**Exitus**	**Surviving**	**Survival ratio**	**Median Survival (MS)**	**Overall survival**	**%95 CI**
Stage	T1	8	3	5	62.5%	32 months	38.83 ± 8.10	22.94–54.72
	T2	9	4	5	55.6%	49 months	57.00 ± 13.05	31.42–82.57
	T3	4	1	3	75.0%	6 months	11.00 ± 3.54	4.07–17.93
	T4	7	4	3	42.9%	12 months	21.57 ± 5.08	11.61–31.53
Stage	N0	20	9	11	55.0%	35 months	53.58 ± 9.34	35.28–71.88
	N1	4	1	3	75.0%	32 months	58.00 ± 18.39	21.97–94.03
	N2	4	2	2	50.0%	8 months	19.00 ± 8.98	1.40–36.60
Surgery type	Lobectomy	13	5	8	61.5%	15 months	54.05 ± 12.24	30.05–78.04
	Pneumonectomy	4	3	1	25.0%	12 months	21.00 ± 6.79	7.69–34.31
	Segmentectomy	4	0	4	100.0%	39 months	41	–
	Extended resection	7	4	3	42.9%	15 months	41.86 ± 13.83	14.75–68.97
Pathology	Adenocarcinoma	19	7	12	63.2%	36 months	60.29 ± 9.40	51.84–78.69
	Squamous cell Ca	5	4	1	20.0%	12 months	12.20 ± 1.36	9.53–14.87
Pathological subtype	Acinar	5	1	4	80.0%	52 months	77.20 ± 13.24	51.2–103.15
	Low differentiated	10	3	7	70.0%	27 months	41.80 ± 7.85	26.41–57.19
	Lepidic	2	1	1	50.0%	12 months	40.00 ± 19.80	1.19–78.81
	Mid differentiated	3	2	1	33.3%	8 months	11.50 ± 3.50	4.64–18.36
Lymphatic invasion	Present	13	6	7	53.8%	26 months	24.26 ± 3.73	16.96–31.56
	Absent	15	6	9	60.0%	32 months	57.13 ± 10.74	36.07–78.18
Visceral pleura invasion	Present	7	4	3	42.9%	8 months	16.50 ± 4.34	7.99–25.00
	Absent	21	8	13	61.9%	45 months	57.86 ± 9.16	39.91–75.81
Cranial therapy	Surgery (+)	16	6	10	62.5%	32 months	52.30 ± 12.00	28.76–75.84
	Surgery (–)	12	6	6	50.0%	15 months	37.54 ± 8.39	21.08–54.00
Cranial met number	1	25	10	15	60.0%	32 months	53.60 ± 8.95	36.06–71.13
	≥2	3	2	1	33.3%	12 months	29.33 ± 15.81	0–60.33
Chemotherapy timing	Neoadjuvant	8	3	5	62.5%	32 months	38.29 ± 9.05	20.54–56.03
	Adjuvant	20	9	11	55.0%	24 months	48.56 ± 10.08	28.81–68.31

**Figure 2:**
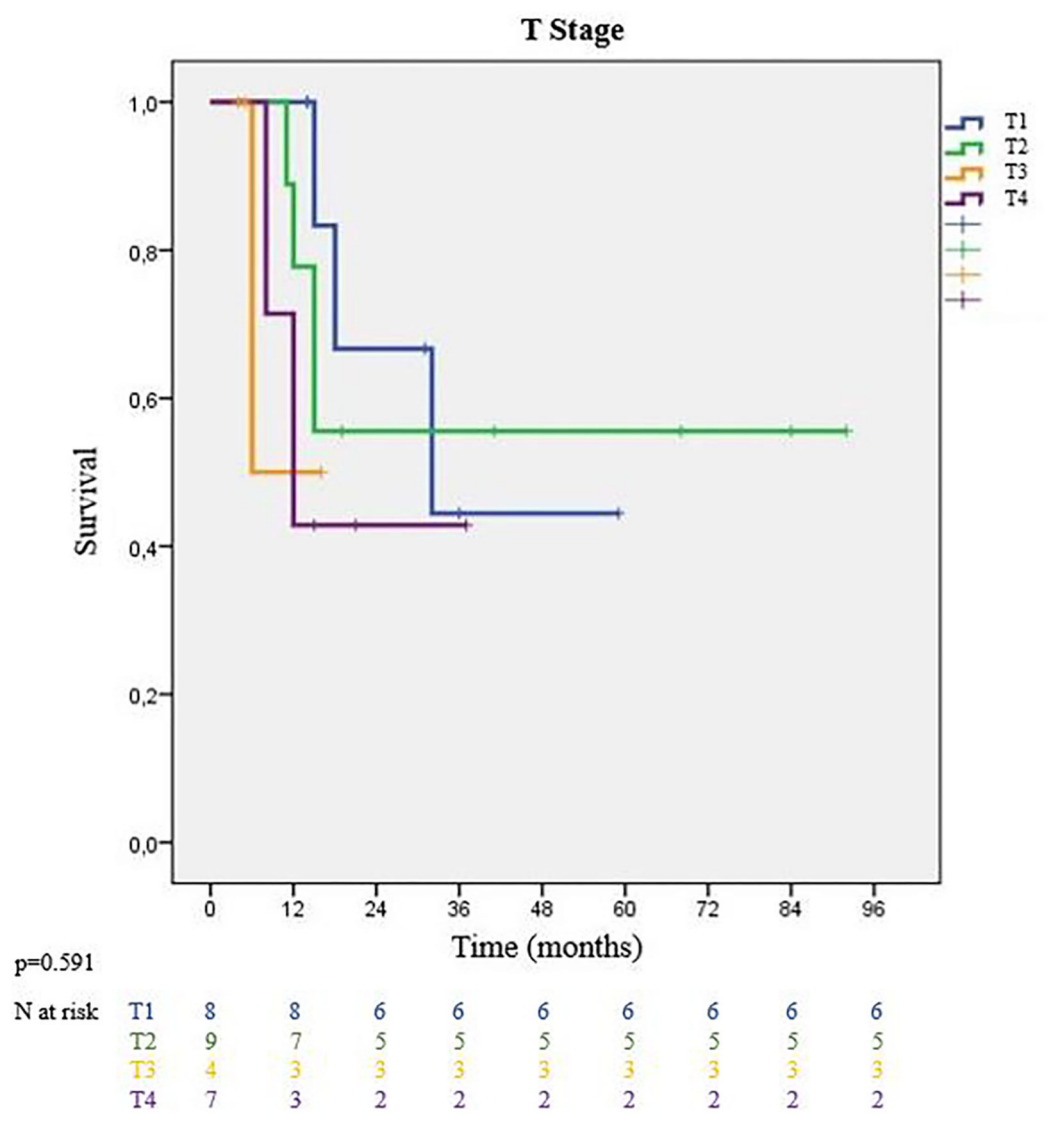
Survival graph according to T Stage.

Intrathoracic staging parameters for lung cancer showed survival changes among groups. The MS for patients with T1 primary tumors was 32 and 49 months for patients with T2 tumors. Larger T3 and T4 tumors ended up with worse MS of 12 and 6 months, respectively (*p* = 0.591) (Figure 2). Additionally, patients who underwent pneumonectomies had worse outcomes, while those with segmentectomies had a 100% survival rate during our follow-up period. Nodal stage evaluation showed that N0 and N1 patients had better median survival than incidental N2 patients (N0: 35 months, N1: 32 months vs. N2: 8 months) (*p* = 0.413). There was no significant survival difference between node negative (N0) and node positive patients (N1-N2 patients combined).

**Figure 3:**
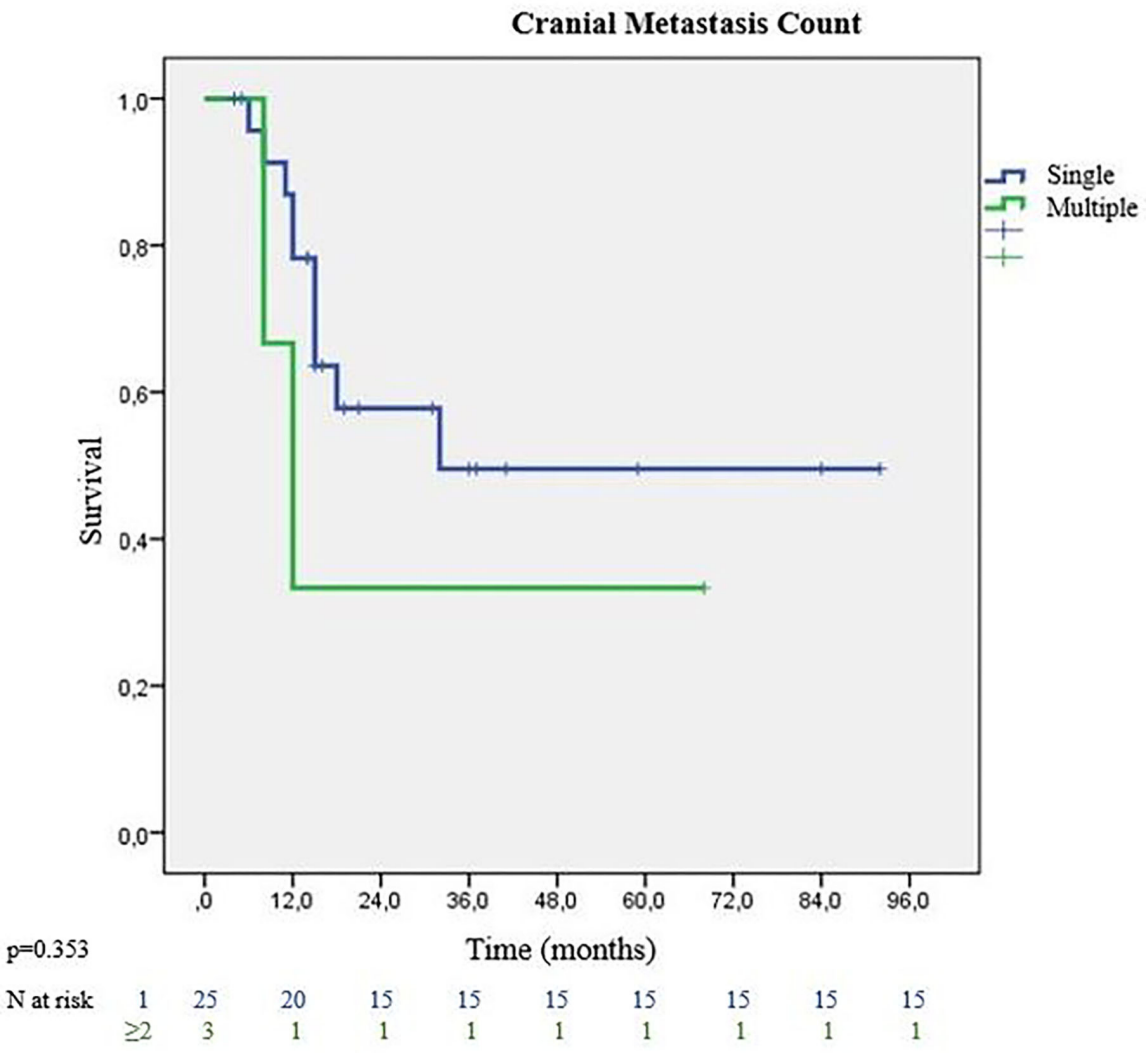
Survival graph according to cranial metastasis count.

All patients except 3 (3/28, %10.7) received surgical treatment of the lung lesion before cranial local treatments. Eight patients (28.6%) were given chemotherapy before pulmonary resection. Patients receiving neoadjuvant chemotherapy showed better MS than the adjuvant group (32 vs. 24 months) (*p* = 0.830). Recurrence or distant metastasis was not detected in 12 (42.9%) patients. Analysis of the treatment types used for cranial metastases, showed that the surgically treated group had better survival rates with MS of 32 months. On the other hand, patients receiving radiotherapy (SRS or WBRT) without surgery, had MS of 15 months (*p* = 0.594). The majority of patients had single cranial metastasis (89.3%). Patients with single metastasis had 32 months MS, while those with multiple metastases had 12 months MS (*p* = 0.353) (Figure 3). On pathological review of the surgical specimens, partial response (PR) to chemotherapy was seen in 11 patients, and stable disease in 17 patients. Complete pathological response was not observed in any of the specimen. Recurrence was observed in 19 patients. The most frequent site of recurrence was a distant site in 11 patients, followed by a cranial site in 5 patients, and local (intrathoracic) site in 3 patients. 3 eligible patients (10.7%) who were programmed death-ligand 1 (PD-L1) positive had immunotherapy at relapses. They survived the follow-up period with 21, 38 and 48 months. None of the patients received targeted therapy.

**Figure 4:**
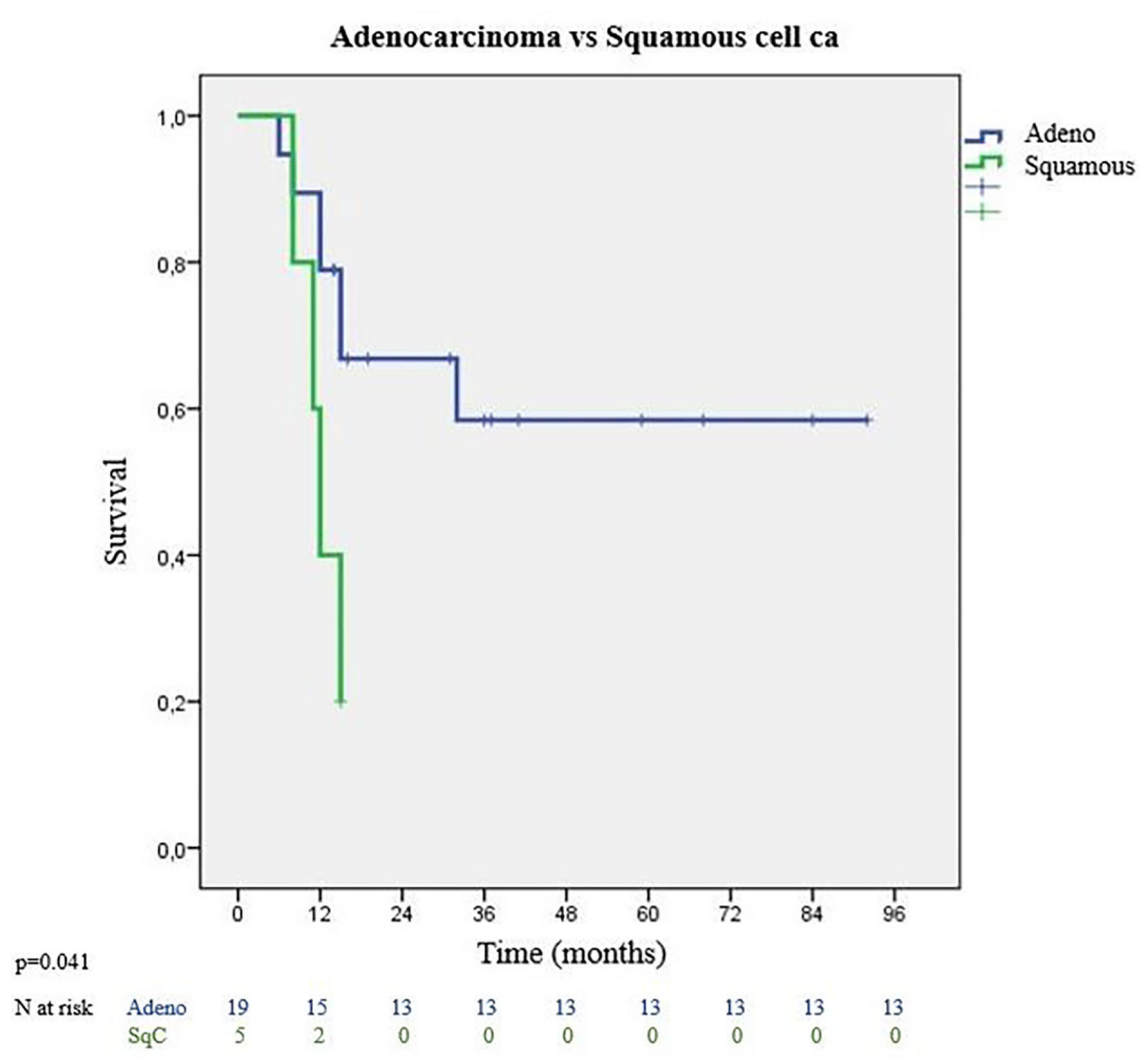
Survival graph according to tumor pathology types.

Tumor characteristics which impacted survival were evaluated with tumor histology, lymphovascular invasion status, and presence of visceral pleura invasion. Most frequent histological type was adenocarcinoma with significantly better survival outcomes. Adenocarcinomas had 36 months MS whereas squamous cell carcinomas had 12 months MS (*p* = 0.041) (Figure 4). Among adenocarcinoma subtypes, acinar adenocarcinoma has the best MS with 52 months, followed by low differentiated adenocarcinoma with 27 months MS (*p* = 0.055). Visceral pleura invasion (MS: 8 months vs. MS: 45 months) (*p* = 0.059) and lymphatic invasion (MS: 26 months vs. MS: 32 months) (*p* = 0.655) of the tumor were found to be negative prognostic factors.

Even though not all of the results are statistically significant, patients having T1-T2 primary lung tumors, no mediastinal lymph node metastasis, receiving minor anatomical lung resection, receiving neoadjuvant chemotherapy, having single cranial metastasis, and receiving surgical cranial metastasectomy were found to have better survival. According to tumor histology, having adenocarcinoma, and not having lymphovascular or visceral pleura invasion correlated with better survival.

## Discussion

Primary therapy for metastatic lung cancer is chemotherapy, achieving 8–11 months MS (2). Retrospective studies of local therapy application to oligometastatic patients showed better outcomes than other stage IV lung cancers (12), and results of recent prospective studies, prove the efficiency of local therapies (surgery, radiotherapy, or both) in oligometastatic patients (13). NCCN guidelines also recommend local aggressive therapy in cranial oligometastatic patients (14). Our study was designed to define the criteria for patients with oligometastatic disease who may benefit from lung resection.

Average survival time in our study is observed as 52.1 ± 8.32 months, and a CS of 60.2% (SD: 9.8%) at first year and 48.3% (SD: %11) at 96 months. These values and ratios for survival are promising, compared with other studies that are designed with similar methodology (8, 15–18) (Table 3). The most remarkable data emerging from our analysis is the median survival of 32 months.

**Table 3:** Survival differences in literature table.

	**Case count**	**5-year OS**
Billing et al. (16)	28	21.4%
Bae et al. (17)	86	22.0%
Yuksel et al. (18)	28	8%
Collaud et al. (8)	19	36.8%
Loi et al. (15)	41	34.4%

When looking at survival analyses according to primary tumor T stage, Casiraghi et al.'s study in 2020 reported that pT1 patients were associated with a significantly better survival rate compared to other T stages (19). In a recently published prospective study, it was reported that a 40% 5-year survival rate can be expected in oligometastatic patients with intrathoracic stage I-II lung cancer (20). In another study that examined the results of patients with solitary cranial metastasis diagnosed as primary lung cancer, and where both cranial lesion and lung lesion were operated on, the median survival of T3 patients was significantly lower than that of T1-T2 patients (18). In our study, we found that the median survival of T1-T2 patients was higher than that of T3-T4 patients. According to our results, pathologic T stage was indeed a prognostic factor of survival. Patients with pathologic pT1 and pT2 tumors had a median survival of 39 months, compared to 12 months for patients with pT3 and pT4 tumors (*p* = 0.181). In conjunction with these data, all of the segmentectomy patients survived the follow-up period. Even though we stated our general principles for lung resection in materials and method for oligometastatic patients, to reduce morbidity and mortality after lung resection keeping them fit for systemic therapies, patients with optimal physiological parameters were chosen even for sublobar anatomical resections.

In studies investigating prognostic factors in oligometastatic disease, it was reported that the presence of mediastinal lymph node metastasis was one of the criteria for poor prognosis (21). In our study, there was no statistically significant difference between the survival rates of 4 patients with incidental mediastinal lymph node metastasis (N2) and 24 patients with N0-N1 disease (*p* = 0.413). This reveals the importance of preoperative correct mediastinal staging. Considering the negative predictive values of non-invasive mediastinal staging methods (such as PET and CT), for lung resection candidates with oligometastatic lung cancer, invasive mediastinal staging is more reliable for accurate mediastinal staging (22).

When looking at studies examining the number of oligometastases as another survival determinant, in the study by Wronski which examined patients with metachronous cranial oligometastasis (200 patients with single metastatic lesion and 31 patients with multiple metastases), it was reported that survival rates were significantly better in patients with single metastasis (23). The authors also stated that 1–3 metastases were associated with better prognosis (24). In our study, statistically significant survival difference was not detected in patients with multiple metastases, although there was observational difference in survival in favor of single metastasis group. Nodal metastasis was not related with multiple cranial lesions. One of the patients with multiple cranial lesions, had incidental N2 metastasis. Only 3 patients with multiple metastases were included in our study; statistically significant survival difference may not have been detected due to the disproportion of patient numbers in the groups.

In our study, adenocarcinoma was the most common histology, which was similar to that reported in the study by Mordant et al. (25). In the study by Bonnette et al. (26), oligometastatic patients with adenocarcinoma histology were reported to be statistically associated with better survival. Adenocarcinoma has different histological patterns with different prognostic values (27). This may suggest that metastases may have more positive biological behavior in cases with solitary metastasis. When we look at the difference in survival among adenocarcinoma pathology subgroups (e.g., acinar, lepidic), the MS of patients in acinar histology was found to be longer than in other histologies. In their 2019 study, Loi et al. (15) reported that lymphovascular and perineural invasion were other variables associated with a negative impact on overall survival. When we examined the survival analyses of patients with visceral pleural and lymphovascular invasion separately, it was found that, although there was no statistically significant correlation between survival rates, the MS of patients with lymphovascular, or visceral pleura invasion was shorter than those without.

Some studies have assumed that circulating tumor cells (CTCs) and microRNAs are another potential method for identifying patients who will undergo local treatment among oligometastatic patients. Krebs and his colleagues found the number of CTCs higher in clinical stage IV non-small cell lung cancer patients than in clinical stage III patients (28). Lussier et al. (29) suggested that expression of certain microRNAs may vary between polymetastatic and oligometastatic patients. In 11 of our 19 patients with recurrence, distant organ metastasis was detected. This may suggest that systemic chemotherapy may be beneficial in these patients. Studies have reported varying results on whether the application of systemic chemotherapy before or after lung surgery is associated with better survival rates. In our study, there was no significant difference between the survival rates of patients who received chemotherapy before or after lung surgery. However, in our algorithm, since the extent of lung surgery may differ, we consider patients' surgical risks of probable, related comorbidities. The patient may not be in a suitable condition to receive adjuvant chemotherapy after surgery, so that a major component of patient's multimodal therapy cannot be given. For locally advanced diseases, we prefer to perform surgery after systemic chemotherapy is initiated to evaluate patients' response to systemic therapy before adding local treatments. It is also useful to discuss the effectiveness of non-surgical treatments in oligometastatic disease. In a study examining the effectiveness of non-surgical treatments for primary tumors performed by Griffioen et al. (24), it was reported that patients undergoing lung cancer surgery showed better overall survival. In a prospective study, De Ruysscyer et al. (13) found 23% overall survival rate of oligometastatic patients who were treated non-surgically. It is difficult to say that non-surgical therapy is more effective according to these results, comparing the survival outcomes of oligometastatic patients treated with surgery.

## Conclusion

Oligometastatic disease in lung cancer is not a rare condition as previously thought. At our institution, among 812 lung cancer patients treated surgically with lung resection, 3.4% were found to have cranial oligometastatic disease. Local therapies should be included to complete multimodal management of the disease. Survival times and ratios that can be reached by including surgery are promising. We suggest that surgical resection in non-small cell lung cancer patients with cranial oligometastatic disease should be a component of the treatment, and when combined with cranial therapy and other oncological treatment methods can result in better survival of patients.

## Data Availability Statement

The raw data supporting the conclusions of this article will be made available by the authors, without undue reservation.

## Ethics Statement

The studies involving human participants were reviewed and approved by Istanbul Demiroglu Bilim University Ethics Committee. The patients/participants provided their written informed consent to participate in this study.

## Author Contributions

EK: conceptualization, methodology, and writing—original draft. EY: writing—original draft. JK: writing—editing. AT: conceptualization, writing—review and editing, and supervision. All authors contributed to the article and approved the submitted version.

## Conflict of Interest

The authors declare that the research was conducted in the absence of any commercial or financial relationships that could be construed as a potential conflict of interest.
